# Medical History from the Earliest Times

**Published:** 1892-04-02

**Authors:** E. T. Withington


### Medical History from the Earliest Times.

By E. T. Withington, M.A., M.B. Oxon.

*(Continued from page 64, Vol. XI.)*

### II.—MEDICINE IN PREHISTORIC TIMES.

Ever since vital activity has existed on the earth, it has, in all probability, been liable to those perturbations which we call disease; wounds and injuries must have occurred wherever there were weapons to inflict them, and fragments of diseased fossil bone are to be found in our museums. We may well suppose that the earliest efforts of intelligent beings were directed towards the avoidance or cure of these disorders, and the older writers amused themselves by searching for instances of medical or surgical practice which they thought might be derived from animals. Thus the most ancient and widely spread of surgical operations, that of bleeding, is said to have been taught mankind by the hippopotamus. That intelligent animal, says Pliny, finding himself plethoric, goes out on the banks of the Nile, and there searches about for a sharp-pointed reed, which he runs into a vein in his leg, and having thus got rid of a sufficient amount of blood, closes the wound with clay. Similarly the use of emetics is said to have been learnt from the dog, that of hellebore from the goat, while the assertion that stags healed their wounds by means of the herb dittany is supported by many authorities, including Aristotle himself. Nor are these stories to be entirely rejected, for have we not lately been told that Count Mattei discovered the basis of his alleged remedies by observing the plants eaten by a mangy dog in the Appennines?

But we are not altogether without indications of a more scientific kind as to the nature of prehistoric medicine, apart from its probable resemblance to the practice of the healing art by barbarous races. Thus philologists assert that among popular medical terms, those denoting affections of the skin are common to the greatest number of branches of the Aryan race, from which we may, perhaps, conclude that such diseases were especially prominent among our uncivilised and not overcleanly ancestors in their earliest homes. So, too, the discovery of cakes made of poppy seed in the ruins of the Swiss lake dwellings may indicate that opium, the most important of drugs, was also one of the earliest to be used by man.

Particularly interesting is the evidence, recently brought forward, of the frequent performance in prehistoric times of the operation of trephining, or removal of a part of the skull vault. At a meeting of the French Association, held in Lyons, 1873, Dr. Prunières, of Toulouse, exhibited a disc of bone, excised from a human skull, which had been found under a dolmen in Lozere. In the following year he showed a number of similar discs, with accompanying skulls, in some of which signs of repair indicated that the patient had long survived the operation though, in the majority, the bone must have been removed either shortly before death or post mortem. Attention was thus directed to the subject, and resulted in the collection of several hundred trephined skulls from prehistoric burial-places in France alone, upwards of sixty being found in one cave, while traces of a similar practice were discovered in countries so remote as Bohemia, Portugal, Peru, and Japan. The accompanying ornaments and weapons were usually those characteristic of the later stone or bronze age, and the operation seems to have been performed by means of flint implements in one of three ways, by sawing, by a chisel-like action, or by boring a series of holes close to one another. In some cases striæ and scratches round the wound betray a bungling operator, but many are done with much skill, considering the rudeness of the instruments.

The various explanations which have been given to account for the frequency of the operation can only be briefly mentioned here. In one or two instances a diseased state of the bone indicates a therapeutic object, and the idea that the operation may have sometimes been performed for the cure of epileptic fits following an injury is supported in a recent paper on the subject by Professor Victor Horsley. But the theory most generally accepted is the one put forward by Broca. That celebrated anatomist and anthropologist noticed that in some cases more than one operation had been performed, one piece of bone having been removed long before death, and one or more others taken from the same skull post mortem, each including part of the healed margin of the old wound. On this he founded his theory that the operation was a sort of religious rite or initiation, the survivors of which acquired a peculiar sanctity, so that, after death, parts of their skulls were sought as amulets, and became so valuable that they were often forged, not only from other human skulls, but from the bones of animals, for similar discs have been found manufactured from a stag's antlers. We might, perhaps, combine these two hypotheses, as, indeed, Broca himself suggested. Epilepsy is, of all diseases, the one most universally attributed to supernatural agencies, and the repeated cure of epileptiform attacks by means of trephining might, not impossibly, have invested the operation with the character of a religious rite, rendering those who underwent it especially proof against the assaults of evil spirits. That the excised fragments of bone were used as amulets is indicated by many of them being perforated, and in the fact that they are found in burial-places Broca saw one of the earliest proofs of belief in a future state, and in a spiritual world where protection against ghostly enemies might be no less necessary than here. Others have supposed that the object of the operation was to enable the spirit to escape from the body, or to facilitate its return, apertures, apparently for this purpose, having been found in many ancient burial mounds. It would thus be connected with the practice, not altogether extinct in modern England, of throwing open the windows of a room where death has recently occurred with the view of favouring the exit of the departed spirit. The discs of bone, it is suggested, might have been worn, not so much as charms against demons as in the hope of acquiring the former possessor's wisdom and cunning, just as a modern savage eats his enemy's heart in order to get his courage.

It is important to notice that a very similar custom still exists in the South Sea. In the island of Uvea, for example, headache and other cerebral disorders are attributed to a crack in the skull or to pressure on the brain, and the recognised treatment is to scrape a hole through the bone near the top of the head. The implement formerly used was a shark's tooth, but since the advent of civilisation broken glass has been substituted. The mortality is about one in two, and few of those who recover are relieved of their symptoms; but such is the force of custom, that there are said to be few adult males on the island who have not undergone the operation.

